# Discharge against Medical Advice at a Teaching Hospital in Ghana

**DOI:** 10.1155/2023/4789176

**Published:** 2023-04-14

**Authors:** Surazu Bayor, Albert Kojo Korsah

**Affiliations:** ^1^Nursing and Midwifery Training College, Zuarungu, Ghana; ^2^Tamale Teaching Hospital, Tamale, Ghana

## Abstract

**Introduction:**

Discharge against medical advice is a global phenomenon where patients voluntarily terminate their consent to medical care before the medical team declares them fit for discharge. The phenomenon adversely affects the delivery of quality health care.

**Methods:**

A retrospective study was conducted at a Ghanaian teaching hospital involving patients who were admitted to the emergency settings within a 2 years period. Data were retrieved from the hospital records and patients discharged against medical advice were identified and studied. Data were cleaned and coded with Excel application and analyzed with SPSS version 23.

**Results:**

A total of 8,565 admissions were made into the ward within the period under review with 210 patients been discharged against medical advice. The prevalence rate was 2.5% with high prevalence seen in male and younger populations. Fractures and head injuries were the commonest conditions for which patients requested to be discharged against medical advice, whilst financial constrains and preference for herbal treatment were the major factors for which patients requested to be discharged against medical advice.

**Conclusion:**

Discharge against medical advice exists and negatively affects the delivery of quality health care in the Ghanaian health sector. Education especially towards at-risk groups such as the younger populations and patients with fractures as well as effective communication between medical team and patients and their families are some proposed measures to reducing the prevalence and negative impacts associated with discharges against medical advice.

## 1. Introduction

Often times, physicians are challenged with patients and family who demands to be discharged home when it is obvious the conditions are not medically fit for discharge [1]. Discharge against medical advice (DAMA) is a global phenomenon with a prevalence rate of more than 2% of all hospital admitted cases according western literature. The prevalence rates may range from 0.3% to 25.9% depending on factors such as geographical history, medical condition, and social status of patients [2]. In comparison, DAMA cases are reportedly higher in developing countries than developed nations. A study conducted in Nigeria reported that, 53.1% of participants requested DAMA within the first 24 hrs of admission [3].

DAMA is defined as the situation where by a patient and family abruptly terminate their consent to treatment and hospitalization before the medical team declares them fit for discharge. The situation usually present as an ethical dilemma to the medical team, considering the patients autonomy to treatment and the medical team's intention to do good [4].

Terminating treatment before being medically fit has unavoidable consequences. Individuals discharged against medical advice forms an at-risk group of both mortality and morbidity [5]. Asthmatic patients who were discharged against medical advice had a four times higher risk of returning to the emergency unit within a month. Most often than not, patients who leave against medical advice return to the health facility with almost always the same illness for which they were initially admitted [6]. In a retrospective study of patients with myocardial infarction (MI), patients who were discharged against medical advice had a 40% higher risk of death or readmission for MI or angina within two years of after discharge.

Several factors compel patients to seek discharge against medical advice. Understanding these factors enables health providers to identify patients at higher risk of seeking DAMA and into intervene appropriately [4]. Cost of treatment, hospital protocol, lack of health insurances, preference of other treatments to modern medicine, fear of amputation, and treatment near home were cited as the major reasons patients seek DAMA [7].

Some prevention strategies to help reduce the prevalence of DAMA have been reported in various literatures. Health providers are advised to formulate management plans that includes the patient in his or her care. Including patients in clinical decisions can help address their extraclinical concerns leading to an agreement with management plan [6]. A 30% decrease in DAMA was reported when a nurse plays the advocacy role for psychiatric patients. The responsibilities of the nurse advocate was to address patients' concerns and fears about their admission [4]. Positive attitudes to clients, reducing waiting time at health facilities and involving social workers have been identified as some factors to reduce the incidence of DAMA [6].

The phenomenon of DAMA has not been adequately explored in Ghana [8], this study therefore seeks to lay the foundation for future studies on discharge against medical advice. The study seeks to explore the prevalence of DAMA in a Ghanaian tertiary health care facility, the at-risk groups for DAMA and the factors that compels patients and families to request for DAMA.

## 2. Methods

### 2.1. Study Design

A descriptive retrospective study was conducted involving a review of admissions into the accident and emergency unit from January 2020 to December 2021. Patients who were discharged against medical advice, children, adults and the aged, were identified and included in the study. However, patients who requested for DAMA and later rescinded on their decision upon counselling from the medical team were excluded from the study.

### 2.2. Study Settings

The study was conducted at the Accident and Emergency unit of the Tamale Teaching Hospital, a Tertiary Health Care referral facility located in Tamale, the administrative capital of the northern region of Ghana. The hospital was established in 1974 to serve as a regional hospital for the northern region. In 2005, the Ministry of Health, Ghana Health Service (GHS) in partnership with the northern regional coordinating council upgraded the facility to a Teaching Hospital, the third in Ghana. The facility serves as the main referral center for the five regions of the north and sections of volta and Ahafo regions [9].

As the third largest tertiary health facility in the country, the hospital has specialized medical, surgical, and gynecological departments. As a teaching hospital, the facility engages in the training of all caliber of health personnel as well as a center for medical research.

The accident and emergency ward is an international standard emergency ward with approximately 50 bed capacity. The accident and emergency ward follows the international triaging system, where patients are categorized and attended to according to the severity of their conditions. The ward is divided into four units, often referred to as zones which includes triage, red, orange, and yellow zone. The triage zone is the unit where patients reporting to the facility are received, attended to, and sorted according to the severity of their conditions. The red zone refers to the resuscitation room for emergency management. Patients admitted into the red zone should obtain a triage score of 7–9. Orange zone refers to the patient waiting area for urgent management. To be admitted into this zone, patients should obtain a triage score of 5-6. The yellow zone refers to the patient waiting area for management. Patients must obtain a triage score of 3-4 to be admitted into this zone [10]. Patients are into the various zones according to the severity of their conditions which is decided by their triage scores. The ward receives cases referred from the various regions of the north and sections of volta and Ahafo regions. The ward manages both adult and paediatric medical and surgical conditions.

The Ghanaian health systems uses the national health insurance system, where subscribers are expected to be provided with some basic services at a cost covered by the insurance providers. However, challenges confronting the insurance system results in subscribers paying out of pocket for services such as medications, laboratory investigation, and treatment [11].

### 2.3. Sample Size Determination

All patients who requested for DAMA at the accident and emergency ward and were discharged against medical advice during the period under review were included in the study. This was obtained by reviewing electronic records that were obtained at the time of granting the DAMA request.

### 2.4. Data Analysis

Data retrieved from the records department of the facility was analyzed. Data was first entered into Excel application, cleaned, coded, and transferred into Statistical Package for Social Sciences (SPSS) version 23 for analysis.

### 2.5. Ethical Consideration

Before the data was retrieved and analyzed, permission was requested from the management of the facility to use the data. Permission was subsequently granted before the analysis proceeded. However, been a retrospective study, the authors lacked the opportunity to seek consent from individual patients as they had already been discharged during the study.

## 3. Results

### 3.1. Participant's Demographics

A total of 8,565 admissions were made into accident and emergency ward within the 2 year period under review. A total of 210 patients requested and were discharged against medical advice, a total of 165 of the patients who were discharged against medical advice were males. This constitutes 78.6% of the total DAMA patients. The female population was 42 patients, representing 20.3% of patients. Most of the patients who were discharged against medical advice were the middle age population. Patient below the ages of 50 yrs were 159, which constitutes a vast 84.5%. The elderly population, those above the age of 50 yrs constitutes the remaining 15.5%.

For educational level, patients whose consent forms were signed were regarded as been educated whilst those whose consent forms were thumb printed were regarded as not educated. In this regard, 33.3% of patients discharged against medical advice were educated whilst 26.2% had no formal education. The results are presented in Table 1.

According to the accident and emergency system, patients are admitted into various wards described by color codes according to the severity of their conditions. The red zones receive patients who require immediate and urgent care as their conditions are severer. The orange zone receives patients with less severe conditions than the red zone, whist the yellow zone receive patients with the least severe conditions among the three. The color codes used are red, orange, and yellow. 25.2% of the patients were admitted to the yellow zone, 63.8% were admitted to the orange zone, whilst 5.2% were admitted to the red zone as illustrated in Table 2.

Majority of the patients 39.0%, prior to been discharged against medical advice have been managed for various degrees and types of fractures. 11.4% have been managed for head injuries, 8.6% for CVA, 4.3% for respiratory conditions such as pneumonia, asthma, and COPD, 8.6% for various degrees of lacerations, and 21.4% for various other conditions such as burns, snake bites, and esophageal cancer. 6.7% of the patients did not have their medical diagnosis documented. The results are shown in Table 3.

Several factors compelled patients to request for discharge against medical advice. Notable among them was financial constraints. This was so widespread that it was categorized according to what patients were constrained to afford, such as medications, investigations, and surgery. Other compelling factors were the preference for herbal treatment, domestic issues, fear of interventions etc. The results are displayed in Table 4.

## 4. Discussions

A total of 8565 patients were admitted within the period under review. The prevalence rate of DAMA was observed to be 2.5%. This prevalent rate is similar to that reported from Nigeria, where 4.7% were reported to have been discharged against medical advice [12]. This may be attributed to the similarities in socioeconomic state of the study settings. In neighboring Ivory coast, however, DAMA prevalence was reported to be as a high as 8.6% [7] This prevalence rate is, however, higher than a similar retrospective study conducted in Saudi Arabia, which reported a DAMA prevalence of 1% [13].

The male gender constituted a larger percentage of those who requested for DAMA. This can be attributed to the fact that, most of the patients were been managed for fractures and head injuries resulting from road traffic accidents (RTAs) involving motorbikes, of which males are mostly victims. Men are challenged with socioeconomic responsibilities of family upkeep. Females are also reported to be more compliant with medical staff than their male counterparts. Similar findings were also reported from a study conducted at an emergency department in Lebanon [14].

Children below the ages of 20 yrs and the aged above 50 yrs constitute 30% of DAMA patients. This may be due to the fact that, children are mostly under parental care, and hence lacks the consent to request for DAMA. The elderly on the other hand are more compliant with medical treatment. Age has been reported to be significantly associated with DAMA [15]. Majority of the patients who requested and were granted DAMA were observed to be educated. Similar findings were also reported from Nigeria [15] and Iran [16].

The mean period of stay within which DAMA was requested was five days on admission. Patients and their families are expected to have exhausted the finances within this period, hence their decision to opt for DAMA.

Prior to their request to discharge against medical advices, majority of the patients were managed for fractures and head injuries. This is attributed to the fact that, the commonest mode of transportation within the Tamale metropolis is motorcycles which mostly results in RTAs. Other notable conditions for which patients were discharged against medical advice were CVA, hypertension, and respiratory conditions such as pneumonia and chronic obstructive pulmonary disease (COPD). Burns, snake bites, electrocution, esophageal cancer, and hemorrhoids were also among the conditions for which patients sought DAMA. These findings agreed with a similar study in Nigeria where 88.5% of patients who were discharged against medical advice were cases of fractures [5]. Studies from Lebanon, however, reported that only 9.8% of patients discharged against medical advice were as a result of trauma, thus fractures [14].

Various reasons were observed to have pushed patients and their families to seek for DAMA. Notable among these compelling factors was financial challenges. Financial constraints have been cited as a major factor for DAMA, especially in African countries [3]. Financial constraints were of varying degrees. Some patients and their families were financially constrained in their abilities to purchase prescribed medications, others were constrained to undergo surgical procedures, whilst others could not afford to undergo various laboratory investigations, yet some other patients were generally constraint and could not afford neither medications nor investigations. Financial constraints was however not a major contributor to DAMA as it only accounted for 7.9% of DAMA cases in Lebanon [14].

Another major factor that compelled patients to request for DAMA was their preference for herbal treatment. Preference for herbal treatment has been reported in DAMA studies involving orthopedic patients [17]. This can be attributed to the general belief that; traditional bone setters are more effective in managing fractures than the modern system of medicine. Despite the level of counselling from the medical team, nothing seems to dissuade patients with fractures from heading to herbal healers. In neighboring Nigeria, 77.1% of patients who took DAMA indicated that, their next destination of care will be herbal medicine practitioners and traditional bone setters [5]. Studies on DAMA from an orthopedic hospital in Nigeria reported that, 62.1% of patients who requested for DAMA indicated traditional bone setters as their next destinations [17].

Other compelling DAMA issues worthy of note includes domestic issues such as the need to take care of families, the need for treatment near home, patients not feeling comfortable in the wards, perceived recovery, perceived poor prognosis, and fear of interventions such as chest tube thoracotomy.

## 5. Conclusion and Recommendations

Discharge against medical advice has a high prevalence in Ghana and its consequences in the delivery of quality health care cannot be overemphasized. The trend which is more prevalent in developing and emerging economies including Ghana adversely affects health care delivery, causing complications and mortalities as well as increasing cost of care. Hospital administrators, clinicians, and other health care workers should make efforts to increase awareness on the consequences of DAMA such as preventable deaths, increased cost, and patient readmissions. Stakeholders in health can develop comprehensive legislations to clinicians and health institutions to regulate the prevalence and consequences of DAMA. Public education on the need to comply with medical care, streamlining the activities of traditional medicine dealers, efficient and effective communication between staffs and patients, and most importantly, providing financial assistance to needy patients are some proposed measures to reduce the impacts of DAMA.

## Figures and Tables

**Table 1:** Patient demographics.

Demographics	Frequency	Percentage (%)
Gender		
Males	165	78.6
Female	42	20.3
Missing	3	1.2
Total	207	98.8

Age		
0–10	17	8.1
11–20	28	13.3
21–30	51	24.3
31–40	43	20.5
41–50	29	13.8
51–60	10	4.8
61–70	8	3.8
71–80	10	4.8
81–90	3	1.4
Missing	11	5.1
Total	199	100

Education		
Educated	70	33.3
Not educated	55	26.2
Missing	85	40.5
Total	210	100

**Table 2:** Various zones at the emergency and their rates of DAMA.

Zone	Frequency	Percentage (%)
Yellow zone	53	25.2
Orange zone	134	63.8
Red zone	11	5.2
Missing	12	5.7
Total	210	100

**Table 3:** Common conditions for which DAMA was requested.

Condition	Frequency	Percentage (%)
Fracture	82	39.0
Head injury	24	11.4
CVA	18	8.6
Lacerations	18	8.6
Respiratory conditions	9	4.3
Others	45	21.4
Missing	14	6.7
Total	210	100

**Table 4:** Factors influencing DAMA.

Factors	Frequency	Percentage (%)
Prefer herbal treatment	59	28.1
Cannot afford investigations	49	23.3
Cannot afford medications	12	5.7
Cannot afford surgery	5	2.4
General financial constrains	20	9.5
Fear of interventions	6	2.9
Domestic issues	5	2.4
Others	9	4.3
Missing	45	21.4
Total	210	100

## Data Availability

The data used in this study are available upon request from Mr. Bayor Surazu.
